# The Dynamics of the Anaerobic Energy Contribution During a Simulated Mass-Start Competition While Roller-Ski Skating on a Treadmill

**DOI:** 10.3389/fspor.2021.695052

**Published:** 2021-07-08

**Authors:** Dionne A. Noordhof, Marius Lyng Danielsson, Knut Skovereng, Jørgen Danielsen, Trine M. Seeberg, Pål Haugnes, Jan Kocbach, Gertjan Ettema, Øyvind B. Sandbakk

**Affiliations:** ^1^Department of Neuromedicine and Movement Science, Centre for Elite Sports Research, Norwegian University of Science and Technology, Trondheim, Norway; ^2^Smart Sensor System, SINTEF Digital, SINTEF AS, Oslo, Norway

**Keywords:** roller skiing, maximal accumulated oxygen deficit method, gross efficiency method, anaerobic capacity, metabolic demand, endurance performance, intermittent exercise, recovery

## Abstract

The purposes of this study were: 1) to investigate the anaerobic energy contribution during a simulated cross-country (XC) skiing mass-start competition while roller-ski skating on a treadmill; 2) to investigate the relationship between the recovery of the anaerobic energy reserves and performance; and 3) to compare the gross efficiency (GE) method and maximal accumulated oxygen deficit (MAOD) to determine the anaerobic contribution. Twelve male XC skiers performed two testing days while roller skiing on a treadmill. To collect submaximal data necessary for the GE and MAOD method, participants performed a resting metabolism measurement, followed by low-intensity warm up, 12 submaximal 4-min bouts, performed using three different skating sub-techniques (G2 on a 12% incline, G3 on 5% and G4 on 2%) on three submaximal intensities on day 1. On day 2, participants performed a 21-min simulated mass-start competition on varying terrain to determine the anaerobic energy contribution. The speed was fixed, but when participants were unable to keep up, a 30-s rest bout was included. Performance was established by the time to exhaustion (TTE) during a sprint at the end of the 21-min protocol. Skiers were ranked based on the number of rest bouts needed to finish the protocol and TTE. The highest GE of day 1 for each of the different inclines/sub-techniques was used to calculate the aerobic and anaerobic contribution during the simulated mass start using the GE method and two different MAOD approaches. About 85–90% of the required energy during the simulated mass-start competition (excluding downhill segments) came from the aerobic energy system and ~10–15% from the anaerobic energy systems. Moderate to large Spearman correlation coefficients were found between recovery of anaerobic energy reserves and performance rank (*r*_*s*_ = 0.58–0.71, *p* < 0.025). No significant difference in anaerobic work was found between methods/approaches (*F*_(1.2,8.5)_ = 3.2, *p* = 0.10), while clear individual differences existed. In conclusion, about 10–15% of the required energy during the periods of active propulsion of a 21-min simulated mass-start competition came from the anaerobic energy systems. Due to the intermittent nature of XC skiing, the recovery of anaerobic energy reserves seems highly important for performance. To assess the anaerobic contribution methods should not be used interchangeably.

## Introduction

Olympic cross-country (XC) skiing competitions range from ~3 min to ~2 h and are completed in different race formats in either the classical or skating technique. The different race formats include interval start races, mass start races, skiathlons, pursuit races, individual sprints, team sprints, and relays (The International Ski Competition Rules (ICR). Book II. Cross-Country., 2020). All these competitions are being organized on varying terrain, resulting in a fluctuating work rate, with supramaximal intensities during uphill sections and submaximal intensities, allowing for recovery, during flat and downhill sections (Sandbakk and Holmberg, 2014). Because of the high power output attained during uphill skiing, these sections are most discriminating between skiers (Sandbakk et al., 2016). The supramaximal intensities reached during uphill sections resulted in an anaerobic energy contribution of ~26% during a roller-skiing sprint time trial of around 3 min, with the anaerobic energy contribution being highly related to sprint performance (Losnegard et al., 2012a). Although the anaerobic energy contribution is in general smaller during longer events (Stellingwerff et al., 2011), it might still be of importance for endurance performance.

Originally, the anaerobic energy contribution is determined during supramaximal constant power output exercise of ~2–3 min (Medbø et al., 1988). However, since the introduction of the maximal accumulated oxygen deficit (MAOD) method, different supramaximal protocols have been used (i.e. constant power output exercise, incremental exercise, all-out exercise etc.) and it seems to be important to choose a protocol that is reflective of the athlete's event (Noordhof et al., 2010). Also as Losnegard (2019) concluded, the energy system contributions during XC skiing races are not solely dependent on the duration of the events, but are also dependent on the varying intensity, as a result of the fluctuating terrain and the pacing pattern adopted. So, to determine the anaerobic energy contribution during skiing races, an exercise protocol with a varying intensity, realized by combining uphill, flat and downhill sections, should be used.

Gløersen et al. (2020) investigated the anaerobic energy contribution during a simulated 13.5-km roller-skiing race on competition terrain. XC skiers repeatedly showed anaerobic energy use, but only a small fraction of their MAOD was expended in these periods, because of the short duration of the different race sections. However, the total anaerobic energy use during the summated sections of active propulsion reached ~380% of participants individual MAOD, from which it was concluded that the recovery and concomitant the ability to repeatedly perform exercise at supramaximal intensities is more important for distance skiing performance, than the anaerobic capacity *per se* (Losnegard, 2019). Gløersen et al. (2020) investigated the anaerobic energy contribution during a simulated individual time trial. However, since 10 out of 12 competitions in international championships are mass-start races, determining the energy demands during mass-start races is of great interest. Therefore, the primary aim of the current study was to investigate the anaerobic energy contribution during a simulated XC-skiing mass-start competition performed while roller-ski skating on varying terrain on a treadmill. In addition, our secondary aim was to investigate the relationship between the recovery of the anaerobic energy reserves and performance. Although, the influence of drafting on the anaerobic energy contribution cannot be taken into account when roller skiing on the treadmill simulated a mass-start race by using a pre-set speed and by letting XC skiers that could not keep up with the set speed take a 30-s rest bout, mimicking them falling back to a chasing group. We hypothesized that a positive relationship between the recovery of anaerobic energy reserves and performance would be found.

Quantifying the anaerobic energy systems contribution is generally done using indirect methods, as direct methods require muscle biopsies and the active muscle mass to be known (Noordhof et al., 2010). The three most commonly used computational methods to determine the anaerobic capacity (i.e., “the maximal amount of adenosine triphosphate (ATP) than can be resynthesized by anaerobic metabolism” (Noordhof et al., 2010)) are the maximal accumulated oxygen deficit (MAOD) method (Medbø et al., 1988), the gross efficiency (GE) method (based on (Seresse et al., 1988)) and the critical power (CP) concept (Monod and Scherrer, 1965). So far, the GE method and MAOD method have been compared during roller-skiing time trials. However, both studies assessed the anaerobic capacity during classical roller-skiing time trials (Andersson and McGawley, 2018; Andersson et al., 2020), while these computational methods have not been applied to skate skiing races. Only in Gløersen et al. (2020) participants were allowed to use different XC skiing (skating) sub-techniques during the simulated race. However, they did not construct separate power output vs. metabolic rate relationships for the different inclines and corresponding sub-techniques to calculate the MAOD but used different inclines to construct one regression line. As the incline and thereby the corresponding sub-technique influences the oxygen cost (Sandbakk et al., 2013), this might have influenced their results. In addition, Gløersen et al. (2020) only determined the anaerobic energy contribution using the MAOD method and did not compare the MAOD and GE method. Therefore, the tertiary aim of the current study was to compare the GE and MAOD method to determine the anaerobic energy contribution during a simulated XC-skiing mass-start competition, while using different skating sub-techniques. To determine the anaerobic energy contribution using both computational methods, the submaximal and supramaximal workloads used were performed on the same inclines.^13^ We hypothesized that large individual differences would be found between methods, comparable as to what has been found during classical roller-skiing time trials (Andersson and McGawley, 2018; Andersson et al., 2020).

## Methods

### Participants

Twelve well-trained (de Pauw et al., 2013) male XC skiers and biathletes (age 25 ± 3 y, height 183 ± 6 cm, body mass 78.9 ± 5.4 kg, peak oxygen uptake ($\overset{∙}{\text{V}}{\text{O}}_{2\text{peak}}$) 69.3 ± 3.8 mL·kg^−1^·min^−1^), competing at a (inter)national level, participated in the current study. The study was pre-approved by the Norwegian Centre for Research Data. Participants were in writing and verbally informed about the experimental protocol and possible risks associated with it, before they provided written informed consent. Participants were asked to abstain from strenuous exercise 24 h before the start of the test day and to prepare like before a competition.

### Experimental Protocol

**Figure 1:**
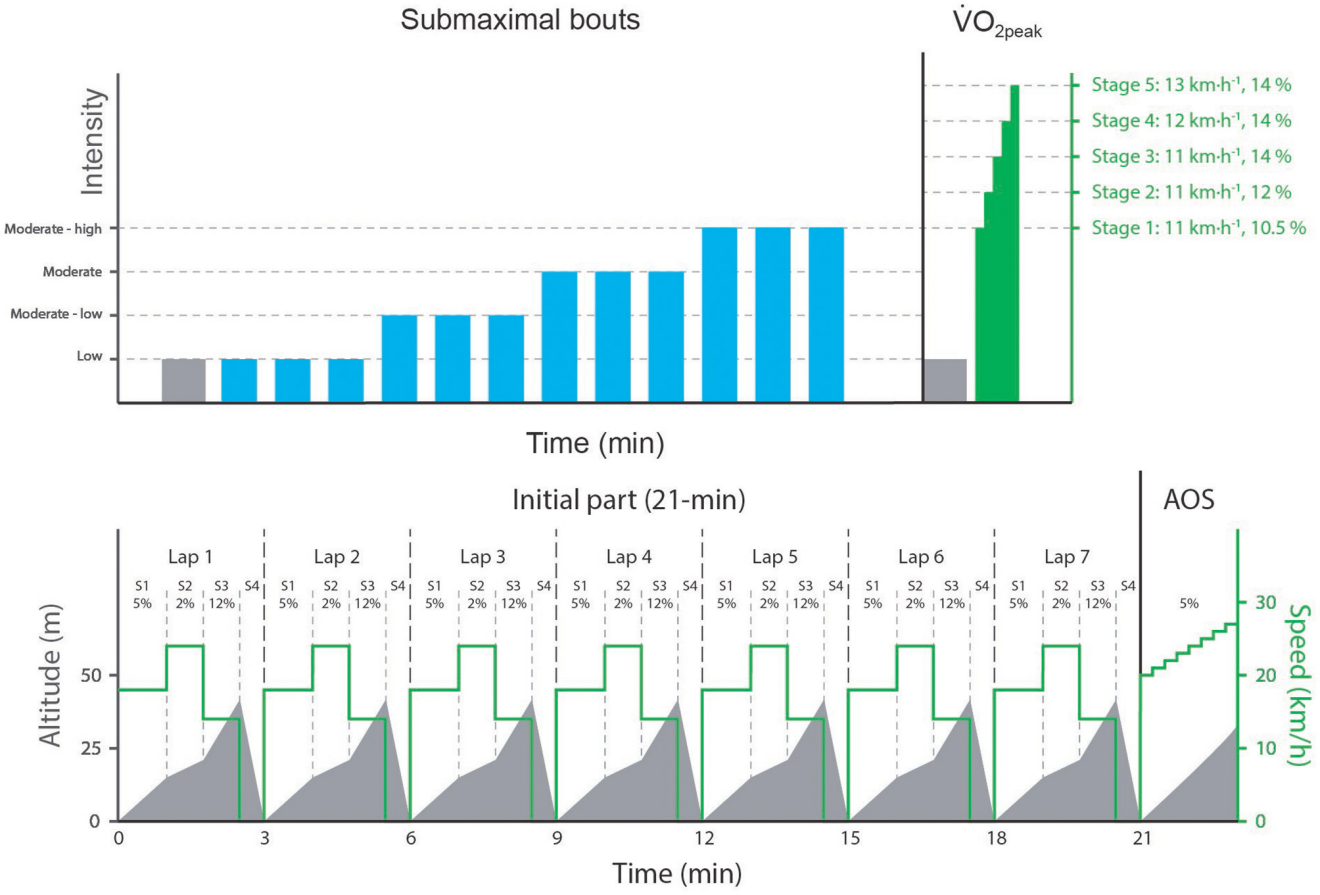
The protocol of the first (upper part of the figure) and second (lower part of the figure) experimental testing day. The three different exercise bouts at the same intensity represent the exercise bouts using either G2, G3 or G4, which were performed in random order. $\overset{∙}{\text{V}}{\text{O}}_{2\text{peak}}$, peak oxygen uptake; S, segment; AOS, all-out sprint. Figure adapted from Seeberg et al. (2021).

Participants completed two experimental testing days. Day 1 was conducted to collect submaximal data necessary for the GE and MAOD method and to assess fitness level. Day 2 was conducted to determine the anaerobic contribution during a simulated XC-skiing mass-start competition. The protocol of the first day (see upper part of Figure 1) consisted of a 4-min measurement of resting metabolism, a 5-min roller-skiing warm up at 10 km·h^−1^ on a 5% incline, followed by 12.4-min submaximal exercise bouts, and a maximal incremental exercise test. Three of the submaximal exercise bouts were performed at a low intensity, while using in random order gear 2 (G2) on a 12% incline (6 km·h^−1^), gear 3 (G3) on a 5% incline (10 km·h^−1^), and gear 4 (G4) on a 2% incline (15 km·h^−1^), three bouts were performed on a moderate-low intensity ((G2 at 7 km·h^−1^, G3 at 12 km·h^−1^, G4 at 18 km·h^−1^), three at a moderate intensity (G2 at 8 km·h^−1^, G3 at 14 km·h^−1^, G4 at 21 km·h^−1^), and finally three submaximal exercise bouts were performed at a high-moderate exercise intensity (G2 at 9 km·h^−1^, G3 at 16 km·h^−1^, G4 at 24 km·h^−1^). These incline-speed combinations were based on pilot testing and previous research (Sandbakk et al., 2012; Grasaas et al., 2014) in between the exercise bouts performed at the three lowest intensities participants rested ~2 min, and in between exercise bouts performed at the highest intensity they rested ~2.5 min. After the last submaximal exercise bouts participants had a 15-min period to rest and actively prepare for the maximal incremental exercise test (see upper part of Figure 1), used to determine $\overset{∙}{\text{V}}{\text{O}}_{2\text{peak}}$.

The protocol of the second experimental testing day (see lower part of Figure 1) has been described before in Seeberg et al. (2021) for a different purpose. The protocol consisted of a 18-min warm up at low to moderate intensity (5 min of G3 at 10 km·h^−1^ on a 5% incline before two 4-min bouts using G2 and G4 (10 km·h^−1^ on an 8% incline)), followed by a 21-min simulated mass-start competition, involving seven identical laps of 3 min, with each lap consisting of four different segments. Participants were familiarized with the mass-start protocol by first completing a 21-min low-intensity version of the course (similar inclines, but lower speeds). Visual and verbal information of the course profile was provided during both simulations. Although, course segments were tailored for specific sub-techniques (due to the speed-incline combination), participants were free to choose sub-techniques, except during the last 30-s segment of each lap performed on a 5% incline (and speed of 20 km·h^−1^). During that section participants had to ski to the front of the treadmill, grab a handle and sit in standardized tuck position (simulated downhill), with a competition-specific knee angle and their elbows resting on their knees, until the next lap started. When participants were unable to keep up with the set speed of the simulated mass-start competition, they could take a 30-s rest bout, by holding on to the handle at the front of the treadmill. Doing this was interpreted as falling back to the chasing group, and thus putting them in a secondary (tertiary or even quaternary) performance group. The simulated mass-start competition ended with a final sprint till exhaustion (see lower part of Figure 1). Participants were encouraged to complete the simulated mass-start competition without non-protocoled rest bouts and to sprint as long as they could.

### Data Collection

At the start of both testing days body mass was determined using an electronic scale (Seca 877, Seca GmbH & Co. KG., Hamburg, Germany). The mass of the equipment was determined using the same scale and was set at 2.6 kg for all participants. Both experimental protocols were performed on a 3-by-5 m motor driven roller-ski treadmill (ForceLink S-mill, Motekforce Link, Amsterdam, the Netherlands). Skiers used their own XC skiing boots, but the same pair of skating roller skis (IDT Sports, Lena, Norway) with an NNN binding system (Rottefella, Klokkarstua, Norway), wheel type 2, and ski poles specific for their body height with carbide tips. The wheels were pre-warmed during the warm-up. The rolling friction coefficient (μ = 0.016) was determined as described previously (Sandbakk et al., 2010) and remained unchanged from before to during and after the experiments.

Speed and incline of the treadmill were checked using the Qualisys system (Qualisys AB, Gothenburg, Sweden). Throughout the maximal incremental test and simulated mass-start competition, participants wore a safety harness attached to an automatic emergency brake. Respiratory data were collected continuously using a computerized metabolic system with a mixing chamber (Oxycon Pro, Erich Jaeger GmbH, Hoechberg, Germany). Gas analyzers and the flow transducer were calibrated according to the manufacturer's guidelines before each test.

A GoPro camera (GoPro Hero 6, GoPro, Inc., San Mateo, CA, USA) was placed behind the treadmill and used to verify sub-techniques and register unforeseen events.

### Data Analysis

Performance was established by the time to exhaustion (TTE) of the final sprint, while skiers were grouped according to the number of rest bouts that they needed to finish the protocol. $\overset{∙}{\text{V}}{\text{O}}_{2\text{peak}}$ was determined as the highest 30-s moving average.

Mechanical power output (PO) was determined by summating the power against gravity and friction (Sandbakk et al., 2013), using the mass of the equipment (2.6 kg) added to the participants body mass. To determine the aerobic and anerobic energy contributions necessary to deliver the mechanical PO, 10-s mixing chamber data were used. Missing 10-s values were replaced by the preceding value, before these data were converted to second-by-second data by giving the previous nine s the same values. In addition, the respiratory data collected during the simulated mass-start competition were synchronized with the PO data, by adjusting for the circulatory transit delay (Barstow and Molé, 1991) from muscles to lungs, using cross-correlation. The time delay was set on 15 s (average 15.08 s), which resulted in the loss of 15 s data at the end of the test.

#### GE Method

To determine the aerobic and anaerobic energy contribution using the GE method, the highest GE attained during the submaximal exercise bouts performed on day 1 of the different inclines/techniques was used and assumed to be constant during the simulated competition. GE was calculated by dividing the mechanical PO by the metabolic rate. The metabolic rate was determined from the average $\overset{∙}{V}O_{2}$ and RER of the final min of the submaximal exercise bouts, using the conversion table of Péronnet and Massicotte (1991) and the thermochemical calorie (1 kCal = 4.184 kJ). The mechanical power aerobically produced (P_aer_) was determined by converting the second-by-second $\overset{∙}{V}O_{2}$ using the conversion table of Péronnet and Massicotte (1991) and the thermochemical calorie (1 kCal = 4.184 kJ), while presuming *RER* = 1.0 when RER is above 1.0 (Foster et al., 2003), and multiplying this value with the incline-specific GE. Subsequently, the mechanical power anaerobically produced (P_an_) can be calculated by subtracting P_aer_ from PO (Seresse et al., 1988; Noordhof et al., 2013). As it is impossible to calculate GE during the simulated downhill, because no PO is delivered, the energy contributions during these segments has not been included. The relative energy contribution was subsequently calculated by summating P_aer_ and P_an_ over the duration of the simulated competition (without and with the final sprint till exhaustion) and expressing this as a percentage of the total work performed.

#### MAOD Method

To determine the energy systems contributions using the MAOD method, a linear PO-metabolic rate (MR) relationships was constructed for each incline/technique (Andersson and McGawley, 2018), based on the average respiratory data of min four (last min) of the day 1 submaximal exercise bouts (MAOD_4−Y_). A second PO-MR relationship was constructed by also including the average individual resting MR as a fixed Y intercept (MAOD_4+Y_), as it has been suggested that this might result in more robust PO-MR(or $\overset{∙}{V}O_{2}$) relationship (Noordhof et al., 2010). The incline-specific regression lines were extrapolated to supramaximal intensities to estimate the required MR (demand) on each second of the competition. The anaerobic MR (MR deficit) was then calculated by subtracting the aerobic (i.e., measured) MR from the required MR. The MAOD was determined by summating the anaerobic MR over the duration of the entire trial, while either excluding or including the simulated downhill segments and either excluding or including the final sprint. The relative energy contribution was subsequently calculated by summating the aerobic MR and the anaerobic MR over the duration of the simulated competition (excluding and including the simulated downhill segments and excluding and including the final sprint) and expressing this as a percentage of the total work performed.

#### Comparing Methods

The energy contributions calculated using both methods were compared by expressing the results of the GE method in metabolic terms instead of in mechanical terms, by using the highest incline-specific GE of day 1.

### Methods – Secondary Experiment

A secondary experiment was conducted to estimate MR during the standardized simulated downhill position, to determine the energy system contributions during the entire simulated mass-start competition and not only the periods of active propulsion, using the MAOD method.

#### Participants

Four male XC skiers (age 31 ± 6 y, height 183.8 ± 2.2 cm, body mass 76.4 ± 9.0 kg), training at a recreational level, participated in the simulated downhill test. Ethical procedures were the same as during the main experiment.

#### Experimental Protocol and Data Collection

The test started with a 10-min roller-skiing warm up at 10 km·h^−1^ on a 5% incline, followed by two 3-min simulated downhill bouts at 20 km·h^−1^ on a 5% incline, while sitting in the standardized tuck position, as explained above. Between the two simulated downhills, participants had 5-min of passive recovery. The same equipment, as described for the main experiment, was used.

#### Data Analysis and Results

Data were analyzed as described above. The average MR during the final minute of both simulated downhills was 310 ± 89.9 W, which was used as the required MR to calculate the anaerobic MR of all twelve participants using the MAOD method (MAOD_4−Y_ and MAOD_4−Y_).

### Statistics

All statistical analyses were carried out using IBM SPSS Statistics (IBM SPSS Statistics 27.0, IBM Corp., Armonk, NY, USA). The aerobic and anaerobic energy systems contributions are presented as mean ± standard deviation (SD). To determine the correlation coefficients between the recovery in the anaerobic energy reserve and performance, participants were ranked based on both variables and Spearman rank-order correlation coefficients were determined. As it was expected that more recovery correlates to a higher performance, a one-tailed test was used. Anaerobic capacity data of all twelve participants were checked for normality by testing the differences in anaerobic capacity between methods (excluding the simulated downhills and including the sprint till exhaustion) using the Shapiro-Wilk test (GE–MAOD_4−Y_ *p* = 0.060; GE–MAOD_4+Y_ *p* = 0.12; MAOD_4−Y_-MAOD_4+Y_ *p* = 0.15). Sphericity was assessed using the Mauchly's test, as the Mauchly's test was significant (*p* = 0.047) and the estimate of sphericity was smaller than 0.75 the Greenhouse-Geisser correction was used. A repeated measures ANOVA was used to test if the three methods (GE, MAOD_4−Y_ and MAOD_4+Y_) resulted in different anaerobic capacities. In addition, the 95% limits of agreement (*mean difference*±*t*_0.975∙*df*_∙*standard deviation of the differences*) were determined to assess the agreement between the three methods (Bland and Altman, 2003). The magnitude of the correlation coefficients and effect sizes were interpreted using the following scale: 0.0–0.1, trivial; 0.1–0.3, small; 0.3–0.5, moderate; 0.5–0.7, large; 0.7–0.9, very large; 0.9–1.0, nearly perfect (Hopkins, 2002).

## Results

Eight skiers finished the simulated mass-start competition without non-protocoled rest bouts, while one skier needed one additional 30-s rest bout, two skiers needed two additional rest bouts, and one skier needed three additional rest bouts to the complete the entire protocol including the final sprint till exhaustion (see Table 1).

**Table 1:** Individual anaerobic work in metabolic terms while including the final sprint till exhaustion and excluding G7, determined using the gross efficiency (GE) method and maximal accumulated oxygen deficit (MAOD) method.

**Participant**	**GE (kJ)**	**MAOD_**4−Y**_ (kJ)**	**MAOD_**4+Y**_ (kJ)**	**Diff GE vs. MAOD_**4−Y**_ (kJ)**	**Diff GE vs. MAOD_**4+Y**_ (kJ)**	**Diff MAOD_**4−Y**_ vs. MAOD_**4+Y**_ (kJ)**	**Additional 30-s rest bouts (n)**	**TTE (s)**
5	202.1	234.1	187.4	−32.0	14.7	46.7	0	130
4	306.9	223.8	291.9	83.1	15.0	−68.1	0	119
6	272.4	267.7	248.6	4.7	23.7	19.1	0	101
11	220.6	202.5	180.5	18.1	40.1	22.0	0	91
12	195.0	215.2	184.6	−20.2	10.4	30.6	0	74
8	323.8	331.2	282.5	−7.4	41.3	48.6	0	65
13	256.2	290.0	191.9	−33.8	64.3	98.1	0	60
1	217.9	228.2	201.4	−10.3	16.5	26.7	0	47
2	263.5	253.7	250.1	9.8	13.4	3.6	1	50
10	245.7	233.3	228.9	12.3	16.8	4.5	2	62
9	258.7	235.3	238.0	23.4	20.6	−2.8	2	47
7	304.0	282.0	244.0	22.0	60.0	38.1	3	66
**Average** **±** **SD**	255.6 ± 41.8	249.8 ± 36.8	227.5 ± 38.3	5.8 ± 31.4	28.1 ± 18.7	22.3 ± 39.1		

*Diff, the difference in anaerobic work between the GE method and one of the MAOD approaches; SD, standard deviation*.

*Participants are ranked based on the number of non-protocoled 30-s rest bouts (the gray shaded rows are data of participants that needed one or more non-protocoled 30-s rest bouts) and time to exhaustion (TTE) in the final sprint*.

### Energy System Contributions During a Simulated Mass-Start Competition

**Figure 2:**
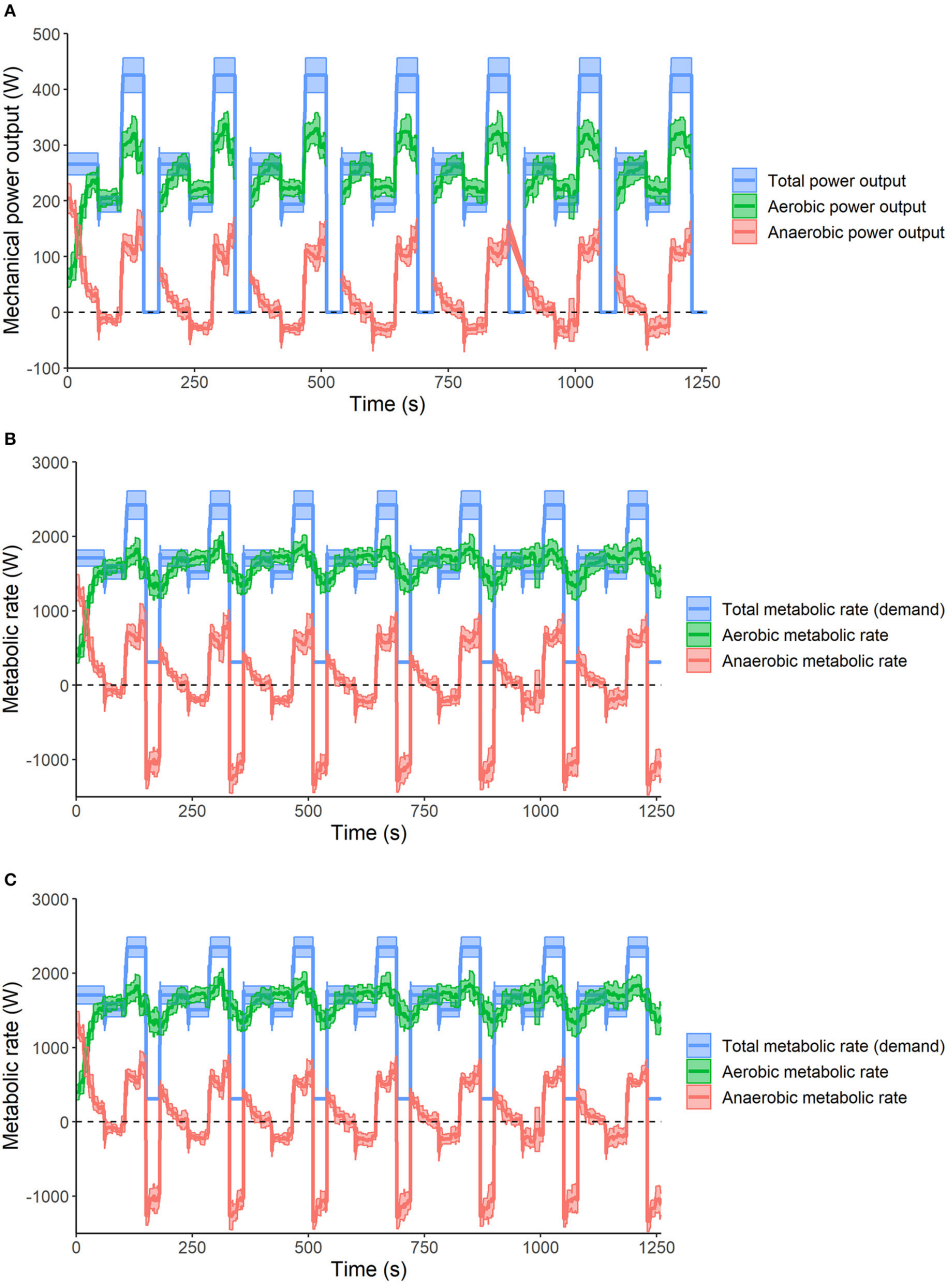
The aerobic and anaerobic energy contributions calculated using the GE method **(A)**, 4-Y MAOD method **(B)** and 4+Y MAOD method **(C)** during the simulated mass-start competition (without the final all-out sprint). **(A)** The aerobic and anaerobic energy contributions have not been determined during the simulated downhills.

The aerobic and anaerobic energy systems contributions calculated using the GE method, MAOD_4−Y_ and MAOD_4+Y_, during the simulated mass-start competition are visualized in Figure 2. Table 2 shows the average accumulated absolute and relative aerobic and anaerobic energy contributions of all twelve skiers and only the eight skiers that completed the protocol without additional breaks.

**Table 2:** The average accumulated absolute **(A)** and relative **(B)** aerobic and anaerobic energy contributions during the simulated mass-start competition. **A)** Absolute energy contribution in mechanical terms for the GE method (kJ) and metabolic terms for the MAOD method (kJ). **B)** Relative energy contributions (%) of both methods.

**Method**	**Aerobic energy (kJ)**	**Anaerobic energy (kJ)**	**Total (kJ)**	**n**
**A**				
GE	269.7 ± 24.4	44.2 ± 6.5	313.9 ± 27.4	12
MAOD4-Y_G7excl._	1793.5 ± 107.2	249.8 ± 36.8	2043.2 ± 127.6	12
MAOD4+Y_G7excl._	1793.5 ± 107.2	227.5 ± 38.3	2021.0 ± 122.5	12
MAOD4-Y_G7incl._	2119.7 ± 102.0	−5.45 ± 39.8	2114.2 ± 123.1	12
MAOD4+Y_G7incl._	2119.7 ± 102.0	−27.7 ± 38.8	2092.0 ± 118.2	12
GE	283.1 ± 17.6	44.3 ± 7.9	327.4 ± 23.3	8
MAOD4-Y_G7excl._	1846.3 ± 85.4	249.1 ± 43.7	2095.4 ± 117.8	8
MAOD4+Y_G7excl._	1846.3 ± 85.4	221.1 ± 46.1	2067.4 ± 120.8	8
MAOD4-Y_G7incl._	2149.6 ± 102.2	10.9 ± 38.4	2160.6 ± 117.8	8
MAOD4+Y_G7incl._	2149.6 ± 102.2	−17.0 ± 41.5	2132.6 ± 120.8	8
^*^GE	263.0 ± 16.7	40.9 ± 7.4	303.9 ± 22.2	8
^*^MAOD4-Y_G7excl._	1717.5 ± 92.6	230.2 ± 44.1	1947.7 ± 130.6	8
^*^MAOD4+Y_G7excl._	1717.5 ± 92.6	202.3 ± 41.3	1919.7 ± 120.5	8
^*^MAOD4-Y_G7incl._	2020.8 ± 107.3	−7.9 ± 38.4	2012.9 ± 130.6	8
^*^MAOD4+Y_G7incl._	2020.8 ± 107.3	−35.9 ± 36.4	1984.9 ± 120.5	8
**Method**	**Aerobic energy (%)**	**Anaerobic energy (%)**	**n**	
**B**				
GE	85.9 ± 1.68	14.1 ± 1.68	12	
MAOD4-Y_G7excl._	87.8 ± 1.39	12.2 ± 1.39	12	
MAOD4+Y_G7excl._	88.8 ± 1.63	11.2 ± 1.63	12	
MAOD4-Y_G7incl._	100.3 ± 1.84	−0.32 ± 1.84	12	
MAOD4+Y_G7incl._	101.4 ± 1.83	−1.38 ± 1.83	12	
GE	86.5 ± 1.69	13.5 ± 1.69	8	
MAOD4-Y_G7excl._	88.2 ± 1.54	11.8 ± 1.54	8	
MAOD4+Y_G7excl._	89.4 ± 1.69	10.6 ± 1.69	8	
MAOD4-Y_G7incl._	99.5 ± 1.74	0.46 ± 1.74	8	
MAOD4+Y_G7incl._	100.5 ± 1.89	−0.47 ± 1.89	8	
^*^GE	86.6 ± 1.68	13.4 ± 1.68	8	
^*^MAOD4-Y_G7excl._	88.2 ± 1.53	11.8 ± 1.53	8	
^*^MAOD4+Y_G7excl._	89.5 ± 1.68	10.5 ± 1.68	8	
^*^MAOD4-Y_G7incl._	100.5 ± 1.89	−0.47 ± 1.89	8	
^*^MAOD4+Y_G7incl._	101.9 ± 1.89	−1.86 ± 1.90	8	

*G7_excl._, accumulated data without the simulated downhill segments; G7_incl._, accumulated data including the simulated downhill segments;*

* *, excluding the all-out sprint data*.

The average absolute and relative contributions of both the aerobic and anaerobic energy systems on the different segments of the course profile are provided in Table 3. All three methods showed that on the relatively flat segments athletes used ~12% more aerobic energy than the demand (~112% in Table 3), which could be used for the recovery of the anaerobic energy reserves (~-12% in Table 3). On the moderate incline about 88% of the energy was generated by the aerobic energy system and ~12% by the anaerobic energy systems, and on the steep incline ~74% was generated by the aerobic energy system vs. ~26% by the anaerobic energy systems.

**Table 3:** The average aerobic and anaerobic energy contributions on the relatively flat segment (2%), moderate incline (5%), steep incline (12%), and simulated downhill segment.

	**GE method**			
	**2%**	**5%**	**12%**	**Downhill**
Aerobic e. (W)	219.2 ± 9.1	233.6 ± 39.9	306.7 ± 15.8	-
Anaerobic e. (W)	−24.8 ± 10.7	31.6 ± 40.3	114.5 ± 18.2	-
Total (W)	194.4 ± 13.5	265.2 ± 7.7	421.2 ± 17.1	0
Aerobic e. (%)	113%	88%	73%	
Anaerobic e. (%)	−13%	12%	27%	
	**MAOD**_**4−Y**_			
	**2%**	**5%**	**12%**	**Downhill**
Aerobic e. (W)	1693 ± 54	1510 ± 258	1755 ± 82	1444 ± 97
Anaerobic e. (W)	−172 ± 77	199 ± 260	649 ± 102	−1134 ± 97
Total (W)	1522 ± 53	1710 ± 13	2404 ± 74	310 ± 0
Aerobic e. (%)	111%	88%	73%	466
Anaerobic e. (%)	−11%	12%	27%	−366
	**MAOD**_**4+Y**_			
	**2%**	**5%**	**12%**	**Downhill**
Aerobic e. (W)	1693 ± 54	1510 ± 258	1755 ± 82	1444 ± 97
Anaerobic e. (W)	−188 ± 77	194 ± 260	581 ± 98	−1134 ± 97
Total (W)	1505 ± 52	1705 ± 15	2336 ± 66	310 ± 0
Aerobic e. (%)	112%	89%	75%	466
Anaerobic e. (%)	−12%	11%	25%	−366

*Data are of the eight athletes finishing the protocol without additional breaks. Contributions are reported absolutely, i.e. in mechanical terms for the GE method and in metabolic terms for the MAOD methods, and relatively*.

### Relationship Between the Recovery of Anaerobic Energy Reserves and Performance

The Spearman correlation coefficients between the recovery of anaerobic energy reserves calculated using the GE method, MAOD_4−Y_ and MAOD_4+Y_ (while excluding the simulated downhills) and performance rank were respectively, 0.58 (*p* = 0.024), 0.71 (*p* = 0.005) and 0.59 (*p* = 0.022), which were considered moderate to large.

### Effect of Computational Method

No statistically significant difference in anaerobic work was found between methods (*F*_(1.2,8.5)_ = 3.2, *p* = 0.10, ω^2^ = 0.08). Although only a trivial effect size was found between computational methods, clear individual differences existed between methods (see Table 1), which is also reflected by the 95% limits of agreement. The 95% limits of agreement between the GE method and MAOD_4−Y_ was 5.8 ± 69.1 kJ, between the GE method and MAOD_4+Y_ 28.1 ± 41.2 kJ and between the MAOD_4−Y_ and MAOD_4+Y_ methods 22.3 ± 86.1 kJ.

## Discussion

The current study is the first to investigate the anaerobic energy contribution during a simulated XC-skiing mass-start competition while roller-ski skating on varying terrain on a treadmill. In addition, we investigated the relationship between the recovery of anaerobic energy reserves and performance and the effect of computational method on the anaerobic energy contribution. The main findings were: 1) on average ~10–15% of the required energy during the simulated mass-start competition (excluding the simulated downhills) came from the anaerobic energy systems and; 2) moderate to large correlation coefficients were found between the (rank in) recovery of anaerobic energy reserves (while excluding the simulated downhills) and performance; 3) although no significant difference in anaerobic work was found between methods, large individual differences existed.

The absolute anaerobic energy contribution in the current study ranged on average from 227.5 to 255.6 kJ for the different methods (see Table 1). The results of the MAOD approaches correspond to 145–149 mL O_2_·kg^−1^, which is considerably lower than the accumulated O_2_ deficit of 299 ± 46 mL O_2_·kg^−1^ found by Gløersen et al. (2020). In both studies the sections of the race without active propulsion (simulated downhills) were excluded from the analysis. The main explanation for this difference is most likely the difference between a time trial vs. a simulated mass-start competition. During the time-trial like XC-skiing skating race simulation in Gløersen et al. (2020), participants could pace themselves optimally and use all their energy reserves to finish the trial as fast as possible (31:48 ± 1:47 [mm:ss]), while participants in the current study were performing a simulated mass-start (21:00 + 1:16 ± 0:28 [mm:ss]) in which the pace was decided for them. Although four participants struggled to keep up with the virtual group and had to take one or more 30-s rest bouts, simulating them to fall back to a secondary, tertiary or quaternary chasing group, eight participants could keep up and might have spent (considerably) less energy than when they would have performed a time trial. Spending less energy results also in a lower anaerobic contribution and therefore explains part of the difference between the current study and Gløersen et al. (2020). Besides, the duration of the time trial in Gløersen et al. (2020) (31:48 ± 1:47 [mm:ss]) was about ten min longer than in the current study (21:00 + 1:16 ± 0:28 [mm:ss]), which most likely also explain a substantial part of the difference in the absolute anaerobic energy contribution. In addition, the participants in the study of Gløersen et al. (2020) had a higher aerobic capacity (77.4 ± 4.4 mL·min^−1^·kg^−1^) than the participants in the current study (69.3 ± 3.8 mL·kg^−1^·min^−1^), with most likely a greater possibility for the recovery of anaerobic reserves, making it feasible to reach a higher anaerobic energy contribution. When expressing the energy contribution relatively, on average ~85–90% of the required energy during the simulated mass-start competition (excluding the simulated downhills) came from the aerobic energy system and ~10–15% from the anaerobic energy systems, which matches with the suggested aerobic energy contribution (85–95%) during distance skiing by Sandbakk and Holmberg (2014). However, when we also include the simulated downhills, we end up with a ~100% contribution from the aerobic energy system.

The recovery of anaerobic energy reserves (while excluding the simulated downhills) was related to performance, which is also what Gløersen et al. (2020) suggested. Based on their results, they concluded that “The ability to recover these energy stores rapidly is therefore likely to be a key performance-determining factor for XC skiers as well as for athletes in other sports with similar demands on bioenergetic systems.” Based on the current results and the results of Gløersen et al. (2020) we agree with Losnegard (2019) that “the “traditionally” held view that the anaerobic energy system plays an insignificant role during distance skiing events seems to warrant re-evaluation”. In addition to a better recovery of the anaerobic energy reserves, also a higher $\overset{∙}{\text{V}}{\text{O}}_{2\text{peak}}$ (moderate correlation coefficient) and thereby the possibility to exercise at a lower relative intensity during the simulated mass start, was associated with performance in the current group of participants (Seeberg et al., 2021). The higher $\overset{∙}{\text{V}}{\text{O}}_{2\text{peak}}$ enabled a lower relative intensity during the first 21 min of the protocol, resulting in more reconstitution of the anaerobic energy expended (Chidnok et al., 2012) and the possibility to continue longer during the final TTE.

When determining the anaerobic energy contribution or anaerobic capacity, different methods can be used (Noordhof et al., 2013). In the current study we compared the GE method and MAOD method, similar as to what has been done before in cycling (Noordhof et al., 2011) and classical XC skiing (Andersson and McGawley, 2018; Andersson et al., 2020). In agreement with Noordhof et al. (2011), we did not find a significant effect of computational method on anaerobic work, while individual differences clearly existed. Although, Andersson and McGawley (2018) and Andersson et al. (2020) found a significant effect of computational method on anaerobic capacity, based on the individual differences and 95% limits of agreement, all three studies (Noordhof et al., 2011; Andersson and McGawley, 2018; Andersson et al., 2020) concluded that different computational methods should not be used interchangeably. Which methods agree most with each other differed between studies, which can most likely be explained by the difference in exercise modality, i.e. XC skiing vs. cycling, and within the XC-skiing studies by the difference in technique [skating in the current study vs. classical skiing in Andersson and McGawley (2018) and Andersson et al. (2020)]. Although the average difference between the GE method and MAOD_4−Y_ is smallest, on an individual basis the difference between these two methods is just as substantial as between the GE method and the MAOD_4+Y_ and between the two MAOD approaches. Finally, it must be mentioned that it remains unclear which method reflects the real anaerobic contribution, as neither the GE method nor the MAOD method has been validated during whole-body exercise (Noordhof et al., 2010; Andersson et al., 2020).

### Strength and Limitations

The current study is the first to investigate the aerobic and anaerobic energy systems contributions during a simulated XC-skiing mass-start competition on varying terrain. Although there are various portable open-circuit spirometry systems available to perform measurements outside in a sport-specific situation, these systems have not been thoroughly validated in cold environments, such as during XC-skiing competitions (Losnegard, 2019). Therefore, measurements were completed in the exercise physiology laboratory, which can be regarded both as a strength and a limitation. By performing all tests in the lab, we could assure high-quality data collected under controlled conditions. However, the ecological validity is smaller compared to a real mass-start competition, where the better-performing skiers would make use of race tactics and drafting, and the different performance groups would determine the intensity themselves, with the secondary, tertiary etc. groups usually having a lower speed than the leading group (unpublished observations).

We used the GE method while assuming a constant GE during the simulated mass-start competition, while it is known that GE decreases during both submaximal (Passfield and Doust, 2000) and (supra)maximal exercise (Noordhof et al., 2015). However, as we did not include submaximal GE measurements after the finish of the simulated mass start and it is currently unclear to what degree GE varies during highly intermittent exercise, we assumed GE to be constant during the race, which most likely resulted in an underestimation of the anaerobic capacity. The same limitation holds for the MAOD method, as the MAOD method assumes a constant PO-MR (or $\overset{∙}{V}O_{2}$) relationship. Finding similar results with the GE method and both MAOD approaches does show the robustness of our findings.

Using the GE method, it is impossible to determine the aerobic and anaerobic contribution during the simulated downhill sections, due to the inability to determine GE when no PO is delivered. Using the MAOD method, it was possible to assess the aerobic and anaerobic energy contribution. However, the MR demand of the simulated downhill was estimated based on respiratory data of a different set of participants, which might have influenced the results.

Lastly, the skiers could freely choose sub-technique during the simulated mass-start, which resulted in the use of mainly G3 on the 12% segments, while the PO-MR relationship and GE assessed on the 12% incline were based on the use of G2. However, it has previously been shown that using G2 and G3 on 6° (~10%) did not result in a significant difference in $\overset{∙}{V}O_{2}$ (trivial effect size) (Losnegard et al., 2012b).

## Conclusions

~10-15% of the required energy came from the anaerobic energy systems during the phases of active propulsion of a simulated mass-start XC-skiing competition. Although the anaerobic energy contribution seems small, moderate to large correlation coefficients were found between the recovery of anaerobic energy reserves and performance. Accordingly, the anaerobic energy contribution and more specifically the recovery of the anaerobic energy reserves seems of importance for distance XC skiing performance. When assessing anaerobic work, different methods/approaches result in large individual differences, indicating that they should not be used interchangeably.

## Data Availability Statement

The data are not publicly available due to privacy concerns. Requests for assessing the dataset should be directed to Dionne A. Noordhof, dionne.a.noordhof@ntnu.no.

## Ethics Statement

Ethical review and approval was not required for the study on human participants in accordance with the local legislation and institutional requirements. The patients/participants provided their written informed consent to participate in this study.

## Author Contributions

JD, GE, PH, JK, DN, ØS, TS, and KS designed the protocol. JD, MLD, PH, DN, and KS conducted the data collection. DN performed the data analyses. MLD, DN, and ØS interpreted the results and DN was responsible for preparing the first draft. All authors revised the manuscript and approved the final version to be published and agree to be accountable for all aspects of the work.

## Conflict of Interest

The authors declare that the research was conducted in the absence of any commercial or financial relationships that could be construed as a potential conflict of interest.
